# Exploring the toxicity of extracts, fractions, and their bioactive components from raw and roasted sea buckthorn seeds on selected human cells

**DOI:** 10.3389/fmolb.2026.1898968

**Published:** 2026-08-11

**Authors:** Natalia Sławińska, Michał Juszczak, Katarzyna Woźniak, Beata Olas

**Affiliations:** 1 Department of General Biochemistry, Faculty of Biology and Environmental Protection, University of Lodz, Łódź, Poland; 2 Department of Molecular Genetics, Faculty of Biology and Environmental Protection, University of Lodz, Łódź, Poland

**Keywords:** antioxidant activity, apoptosis, cytotoxicity, genotoxicity, sea buckthorn seeds

## Abstract

**Introduction:**

With cancer being one of the leading causes of death worldwide, research into new chemical compounds that could be used in its prevention and treatment is of utmost importance. Plants are rich in diverse active compounds and can be a source of effective anticancer agents. Sea buckthorn (*Hippophae rhamnoides* L.) is a promising plant that shows anti-proliferative potential; however, its molecular mechanisms remain unclear. Isorhamnetin glycosides (including isorhamnetin 3-*O*-β-glucoside-7-*O*-α-rhamnoside) are major components of sea buckthorn seeds; multiple studies showed that isorhamnetin possesses anticancer properties, however, the activity of its many derivatives remains unclear. On the other hand, sea buckthorn seeds also contain serotonin (5-HT), which might stimulate the growth of several types of cancer.

**Methods:**

Our present study assessed the *in vitro* cytotoxicity and genotoxicity of two extracts (from raw and roasted seeds), seven fractions (three fractions (a, b, and c) from raw sea buckthorn seeds and four fractions (d, e, f, and g) from roasted sea buckthorn seeds), and two chemical compounds (isorhamnetin 3-*O*-β-glucoside-7-*O*-α-rhamnoside, and serotonin) toward PBMC (peripheral blood mononuclear cells), HL-60 (human acute promyelocytic leukemia), and A549 (human lung adenocarcinoma) cells. Their pro-apoptotic potential, toxicity toward blood platelets, and effect on ROS generation were assessed as well.

**Results and discussion:**

Fraction b (containing mostly flavonol glycosides) had the most promising anticancer activity out of all the tested samples – at the concentration of 50 μg/mL, it decreased the viability of HL-60 cells through the induction of apoptosis. At the same time, it was safe for PBMC and blood platelets. On the other hand, fraction c (that contained serotonin, B-type proanthocyanidins, and other compounds) stimulated A549 cells and, therefore, should be treated with caution. Fractions from roasted seeds were overall weaker than fractions from raw seeds, suggesting that thermal processing might have reduced their activity. In conclusion, fraction b from raw sea buckthorn seeds showed anticancer potential against HL-60 cells, but it should be studied further to determine the activity of individual compounds contained within, their mechanisms of action, and efficiency *in vivo*.

## 1 Introduction

With cancer being one of the leading causes of death worldwide, research into new chemical compounds that could be used in its prevention and treatment is of utmost importance. Lung cancer is one of the most common types of cancer; approximately 85% of lung cancer cases are classified as non-small cell lung cancer (NSCLC). It has a high metastasis potential, which leads to only 15% survival 5 years after diagnosis (Tu et al., 2022). Acute promyelocytic leukemia is a subtype of acute myeloid leukemia. It is currently considered curable; it has a high percentage of early death within 30 days of diagnosis (4%–26%), which is mostly caused by severe bleeding events (Hisada, 2024).

Blood platelets play a significant role in cancer. It is a well-known fact that cancer patients have an increased risk of venous thromboembolism, which occurs in up to 1 in 5 patients. Elevated platelet counts are linked with poor prognosis in several types of cancer, including lung, gastric, or ovarian cancers. Platelets promote hematogenous metastasis; some cancer types release platelet agonists such as adenosine diphosphate or thromboxane A_2_, leading to their activation and platelet-tumor adhesion. Some of the mechanisms of platelet-driven metastasis include shielding cancer cells from shear stress and preventing their recognition by natural killer (NK) cells. Due to this, antiplatelet compounds are being investigated for their usefulness in cancer treatment, with the hope of developing new therapies that carry a lower risk of bleeding. Anticoagulant medication is routinely used in the treatment of thromboembolism, but its use in cancer patients is associated with much higher bleeding risks than in non-cancer patients. Moreover, patients with hematological malignancies (for example, acute promyelocytic leukemia) often suffer from thrombocytopenia, which further increases the bleeding risk. On the other hand, platelets can also play a positive role in cancer by restricting angiogenesis, hindering leakage, or preventing intravasation and extravasation (Josefsson, 2025; Mantha et al., 2018; Tao et al., 2021). Our previous research showed that sea buckthorn seeds have antiplatelet potential, but their effect on cancer cells has not been studied extensively (Sławińska et al., 2024a; Sławińska et al., 2023). For example, our earlier results demonstrated that extracts from raw and roasted sea buckthorn seeds decrease the exposition of the active form of GPIIb/IIIa on the surface of blood platelets activated with 10 μM ADP and 20 μM ADP. In addition, the extract from raw seeds decreased the exposition of P-selectin on the surface of platelets stimulated with 20 μM ADP (Sławińska et al., 2024a).

Plants are rich in diverse active compounds and can be a source of effective anticancer agents. For example, paclitaxel (a broad-spectrum drug used in the treatment of ovarian, lung, or breast cancer) was first isolated from *Taxus brevifolia* (Zhu et al., 2025; Sharifi-Rad et al., 2021). Different parts of sea buckthorn also showed anti-proliferative, proapoptotic, and immunomodulatory activity; however, their molecular mechanisms of action remain unclear (Olas et al., 2018). Sea buckthorn (*Hippophae rhamnoides* L.) is a thorny, deciduous tree or shrub with orange berries. It belongs to the Elaeagnaceae family and is native to temperate regions of Europe and Asia. Currently, it is distributed in 52 countries around the world and cultivated mainly in China, Russia, Finland, Germany, and Estonia. Various sea buckthorn products (e.g., oil, juice, jelly, tea, wine, yoghurt, or bread) are available on the market (Ma et al., 2023; Wang Z. et al., 2022; Zhu et al., 2025).

Sea buckthorn is known for its rich phytochemical content and is estimated to contain around 200 bioactive compounds with different functions, including not only anti-cancer, but also antioxidant, antiplatelet, anti-inflammatory, immunomodulatory, antimicrobial, hepatoprotective, neuroprotective, and wound-healing activities (Abdullahzadeh and Shafiee, 2021; Juszczak et al., 2021; Ma et al., 2023; Olas et al., 2018; Sławińska et al., 2024a; Smida et al., 2019; Xu et al., 2024; Zhu et al., 2025). Sea buckthorn seeds are studied less often than the whole berries, oil, or leaves. They contain flavonoids (mostly glycosides of isorhamnetin, quercetin, and kaempferol), phenolic acids, proanthocyanidins, and triterpenoid saponins, among others (Arimboor and Arumughan, 2012; Chen et al., 2014; Danielski and Shahidi, 2025; 2024; Sławińska et al., 2024b). Previous studies have also shown that sea buckthorn seeds contain serotonin (5-HT), which carries out crucial biological functions in both animals and plants (Galitsyn et al., 2023; Negri et al., 2021; Sławińska et al., 2024b). Multiple studies reported that serotonin might stimulate the growth of several types of cancer (including lung cancer), so the effect of this compound on the human body must be considered carefully (Balakrishna et al., 2021; Tu et al., 2022).

Isorhamnetin and its glycosides (including isorhamnetin 3-*O*-β-glucoside-7-*O*-α-rhamnoside) are major components of sea buckthorn seeds. Various studies have shown that isorhamnetin possesses anticancer properties (Gong et al., 2020; Lei et al., 2024). For example, it inhibited the progression of ovarian cancer (Wang M. et al., 2022), suppressed the growth of gastric cancer cells (Li et al., 2022), and showed anti-proliferation activity toward lung cancer both *in vitro* and *in vivo* (Li et al., 2015; Ruan et al., 2015). Other major flavonoids found in sea buckthorn – kaempferol and quercetin – also showed anticancer activity (Guo et al., 2021; Kundrapu and Malla, 2023; Nandi et al., 2023; Pu et al., 2024; Qattan et al., 2022; Xu et al., 2024).

Thermal processing, which is often applied to seeds during food preparation, can have varied effects on the content of phytochemicals and their activity. High temperature can degrade compounds into less active forms or transform them into derivatives with stronger biological activity. Heat treatment can cause polymerization or release compounds from the food matrix, increasing their bioavailability (Liu et al., 2024a; Narra et al., 2024). Phytochemical analysis of three fractions (a, b, and c) from raw sea buckthorn seeds and four fractions (d, e, f, and g) from roasted sea buckthorn seeds was performed in our earlier studies. LC–MS analyses showed that serotonin is the dominant constituent of fractions c and f, which were tentatively identified on the basis of their HRMS and UV spectra. Moreover, fractions c and f, as well as b and e, contained different proanthocyanidins. Fractions b and e consisted mostly of numerous glycosides of kaempferol, quercetin, and isorhamnetin (Sławińska et al., 2024b).

Studies have shown that thermal processing can increase or decrease the cytotoxic properties of plant material by modifying its chemical content (Chen et al., 2023; Kowalczewski et al., 2019; López et al., 2022). Interestingly, our earlier results indicate that the fractions from roasted sea buckthorn seeds (d and g) are more effective antiplatelet factors than fractions from raw sea buckthorn seeds (Sławińska et al., 2025). Our results of oxidative stress assays (in human plasma treated with an oxidative stress inducer (Fe^2+^/H_2_O_2_ – the donor of hydroxyl radicals)) also demonstrated that fraction g (isolated from roasted sea buckthorn seeds and containing mainly dihexoses and serotonin) had antioxidant properties (Sławińska et al., 2024b).

This study assessed the cytotoxicity and genotoxicity of two extracts (from raw and roasted seeds), seven fractions (three fractions (a, b, and c) from raw sea buckthorn seeds and four fractions (d, e, f, and g) from roasted sea buckthorn seeds), and two chemical compounds (isorhamnetin 3-*O*-β-glucoside-7-*O*-α-rhamnoside (a major component of fraction b), and serotonin (present in fraction c)) toward PBMC (peripheral blood mononuclear cells), HL-60 (human acute promyelocytic leukemia), and A549 (human lung adenocarcinoma) cells. Their *in vitro* pro-apoptotic potential, toxicity toward blood platelets, and effect on ROS generation were assessed as well. Importantly, the concentrations used in our present experiments (≤50 μg/mL) are usually achievable athrough oral administration of phenolic compounds (Manach et al., 2004; Manach et al., 2005; Johnson, 2007).

## 2 Materials and methods

### 2.1 Chemicals

Serotonin was purchased from Thermo Fisher Scientific (Waltham, Massachusetts, USA). Methanol (isocratic grade), TRIS, agarose, DAPI (4′,6-diamidino-2-phenylindole), 2′,7′-dichlorofluorescin diacetate (H_2_DCFDA), camptothecin, and resazurin sodium salt were acquired from Merck (Darmstadt, Germany). The kit for measuring LDH activity was from Cayman Chemical (Ann Arbor, USA). The kit for measuring apoptosis (ApoTox-Glo Triplex Assay) was purchased from Promega (Madison, USA). Cell culture media (RPMI 1640, IMDM, and DMEM-F12), Fetal Bovine Serum (FBS), Phosphate-Buffered Saline (PBS), Lymphosep, Trypsin, Hanks' Balanced Salt Solution (HBSS), and antibiotics (100 x penicillin/streptomycin solution) were from Biowest (Nuaillé, France). All the other reagents were purchased from commercial suppliers, including POCH (Avantor Performance Materials, Gliwice, Poland) and Chempur (Piekary Śląskie, Poland).

### 2.2 Plant material

Plant material was previously described by Sławińska et al. (2024a). Sea buckthorn fruits were collected from a horticultural farm in Sokółka, Podlaskie Voivodeship, Poland (53° 24′ N, 23° 30′ E). The plant material was identified by Mr. Stanisław Trzonkowski, the plantation owner. Whole branches with ripe fruits were harvested in the second half of August 2017. A voucher specimen (IUNG/HRH/2017/1) was deposited at the Department of Biochemistry and Crop Quality, Institute of Soil Science and Plant Cultivation, State Research Institute, Puławy, Poland. The fruit was picked manually and frozen. To isolate the seeds, the fruit was homogenized in water, using a kitchen blender. The skins and pulp were washed away with tap water, while the sedimented seeds were rinsed with methanol to facilitate drying, air-dried, and stored at room temperature.

A portion of sea buckthorn seeds was subjected to thermal treatment in a bakery firm, as described before (Sławińska et al., 2023). The applied roasting method was chosen to imitate the conditions used in the baking of rye bread. The temperature in the baking chamber at the beginning (approx. 10 min) was 280 °C, and the relative humidity was 35%. In the second phase, the temperature was lowered to 175 °C. The total roasting time was 36 min.

### 2.3 Preparation of sea buckthorn seed extracts, fractions, and their phytochemical characteristics

Isolation of the extracts was previously described in Sławińska et al. (2023). Sea buckthorn seeds were milled in a coffee grinder, defatted with hexane at ambient temperature, and dried. 282.4 g of defatted seeds were extracted with 2.5 L of 80% methanol (*v*/*v*) (5 h, ambient temperature), then with 2.5 L of methanol (same conditions). The filtered extracts were pooled, defatted by liquid-liquid extraction with hexane, and rotary evaporated to remove organic solvents. The residue was diluted to a volume of about 400 mL with Milli-Q water and subjected to liquid-liquid extraction with butanol (4 × ∼100 mL). The butanolic extract was rotary evaporated to dryness, dissolved in 20% *tert*-butanol in Milli-Q water, and freeze-dried (Gamma 2–16 LSC, Christ, Osterode am Harz, Germany), to yield 1.566 g of the seed extract. Roasted sea buckthorn seeds were milled in a coffee grinder, defatted with hexane at ambient temperature, and dried. The dried material (91 g) was subjected to a four-step ultrasound-assisted extraction in an ultrasonic bath: 500 mL 80% methanol (*v*/*v*; 30 min), 400 mL 80% methanol (30 min), 300 mL 80% methanol (30 min), and 200 mL 100% methanol (20 min). The filtered extracts were pooled, defatted by liquid-liquid extraction with hexane, and rotary evaporated to remove organic solvents. Next steps of the extract preparation were the same as described above. Finally, 1.930 g of the extract was obtained.

UHPLC-MS analyses of the sea buckthorn seed extracts were performed using an ACQUITY UPLC system (Waters Inc., Milford, MA, USA), equipped with a PDA detector and a triple quadrupole mass detector (TQD; Waters). This procedure was described extensively in Sławińska et al. (2023).

To obtain the fractions, the extracts were separated by reversed-phase liquid chromatography; the fractionation procedure is described in Sławińska et al. (2024b). Fractions a, b, and c were prepared from raw seed extract and contained compounds eluted with 80%, 66%, and 20% methanol, respectively. Factions d, e, f, and g were obtained from roasted sea buckthorn seed extract and contained compounds eluted with 85%, 66%, 20%, and 1.5% methanol, respectively. The composition of the investigated fractions was analyzed by UHPLC-ESI-HRMS/MS, using a Thermo Ultimate 3000RS (Thermo Fischer Scientific, MS, USA) chromatographic system, equipped with a charged aerosol detector (CAD) and coupled with a Bruker Impact II Q-TOF mass spectrometer (Bruker Daltonics GmbH, Bremen, Germany). Fraction a contained multiple triterpenoid saponins, mostly acylated with (2*E*)-2,6-dimethyl-6-hydroxy-2,7-octadienolic acid. The content of fraction d was very similar to that of fraction a. Apart from triterpenoid saponins, fractions a and d also contained triterpenoids, lysophospholipids, putative hydroxylated fatty acids (oxylipins), traces of glycerophospho N-acylethanolamines, and small amounts of flavonoids. Fraction b contained diverse flavonol glycosides, mostly 3-*O*-dihexoside-7-*O*-deoxyhexosides of kaempferol, isorhamnetin, and quercetin. Isorhamnetin 3-*O*-glucoside-7-*O*-rhamnoside was another major constituent of fraction b; its peak constituted about 4.3% of the total peak area (CAD). Fraction e was very similar to fraction b. Fractions b and e also contained small amounts of compounds acylated with phenolic acids (sinapic, ferulic acid, or (2*E*)-2,6-dimethyl-6-hydroxy-2,7-octadienolic acid (*E*)-linalool-1-oic acid), dimeric proanthocyanidins, epicatechin, catechin, (epi)gallicatechin, phenolic acid derivatives, alkaloids (for example, tentatively identified harmalol and harmol), sesquiterpenoid hexosides, several triterpenoid saponins, and a vomifoliol glycoside. Peaks of acylated flavonoids in fraction e were generally lower than those of fraction b. Serotonin was the dominant compound of fraction c; the peak of serotonin (also containing a few co-eluting compounds, present in much smaller amounts) constituted about 28.6% of the total peak area (CAD) of this fraction. Fraction c also contained different B-type proanthocyanidins (dimers and trimers of (epi)catechin, and/or (epi)gallocatechin), putative nucleosides (uridine, guanosine, xanthosine), aromatic amino acids (phenylalanine, tryptophan), and methylgallic acid. The composition of fraction f was similar to that of fraction c, but they differed in the content of some compounds, such as sulfur-containing putative dipeptide and a putative indolylacetic acid hexoside. Fraction g consisted of polar compounds, mainly dihexoses. It also contained a small amount of trihexoses, a dihexose derivative, and serotonin (the peak of the dihexose derivative and serotonin constituted less than 4.3% of the total peak area (CAD) of the fraction). A full description of the phytochemical analysis of the fractions can be found in the article by Sławińska et al. (2024b).

### 2.4 Isolation of isorhamnetin 3-O-β-glucoside-7-O-α-rhamnoside

The procedure of isolation of isorhamnetin 3-*O*-β-glucoside-7-*O*-α-rhamnoside was described in detail by Żuchowski et al. (2019).

### 2.5 Preparation of stock plant extracts, fractions, and their bioactive compounds

Sea buckthorn extracts, fractions, and serotonin were dissolved in 5% DMSO, and isorhamnetin 3-*O*-β-glucoside-7-*O*-α-rhamnoside in 75% DMSO. The final concentration of DMSO in the samples was below 0.05% (*v*/*v*) (for the fractions) and 0.75% (*v/v*) (for isorhamnetin 3-*O*-β-glucoside-7-*O*-α-rhamnoside). 0.05% and 0.75% DMSO were added to the vehicle control samples in each experiment. Low concentrations of DMSO do not affect cell viability (data not presented).

### 2.6 Blood samples

Human whole blood for platelet isolation was collected at the “Diagnostyka” blood collection center located at Brzechwy 7A Street, Lodz, Poland. All donors were healthy, non-smoking volunteers who did not drink alcohol or take any supplements or medication for 2 weeks before blood collection. Blood was drawn into tubes with citrate/phosphate/dextrose/adenine (CPDA) anticoagulant and immediately transported to the laboratory.

The analysis of blood samples was performed according to the guidelines of the Helsinki Declaration for Human Research. All participants signed an informed consent form 1 day before blood collection. Procedures were conducted with the consent of the Bioethics Committee at the University of Lodz (approval code: 2/KBBN-UŁ/III/2014).

Leucocyte buffy coats for peripheral blood mononuclear cells (PBMC) isolation were purchased from Blood Bank (Franciszkańska 17/25 Street, Lodz, Poland) and were used on the same day. The research protocol involving human peripheral blood mononuclear cells (PBMCs) was approved by the University of Lodz Research Ethics Committee (protocol code: 12/KEBNUŁ/I/2024-2025) on 17 December 2024.

### 2.7 Blood platelet isolation

Blood platelets were isolated from whole blood by differential centrifugation. CPDA-anticoagulated full blood was centrifuged at 235 × g for 12 min. Then, platelet-rich plasma was collected and centrifuged at 1,020 × g for 15 min. The pellet was gently reconstituted in Barber’s buffer (a modified Tyrode’s buffer; 0.014 M Tris, 0.14 M NaCl, 5 mM glucose, pH 7.4). The number of platelets was determined by a spectrophotometric measurement (Spectrophotometer UV/Vis Helios alpha; Unicam, Cambridge, UK) at 800 nm and adjusted to 2.0 × 10^8^/mL (Lis et al., 2019; Walkowiak et al., 1997).

### 2.8 PBMCs isolation

PBMCs were isolated from leucocyte buffy coats. First, blood was diluted 1:1 with PBS (pH 7.4). All subsequent procedures were carried out according to the product manual for Histopaque-1077 (Merck, Darmstadt, Germany), a density gradient suitable for PBMC isolation. The diluted blood was layered on Histopaque-1077 and centrifuged at 400 × g for 30 min at room temperature. The fraction containing PBMC cells was collected and washed three times with PBS. The supernatant was discarded, and the cell pellet was suspended in RPMI 1640 medium containing 10% FBS and penicillin/streptomycin solution (100 U/mL and 100 μg/mL, respectively).

### 2.9 Preparation of HL-60 and A549 cells

The human promyelocytic leukemia (HL-60) and the human non-small-cell lung cancer (A549) cell lines were obtained from the American Type Culture Collection (ATCC). Cells were cultured in flasks in a CO_2_ incubator (37 °C, 5% CO_2_). HL-60 cells were cultured in Iscove’s Modified Dulbecco’s Medium (IMDM) containing 2 mM L-glutamine, 25 mM HEPES, 15% inactivated fetal bovine serum (FBS) and penicillin/streptomycin solution (100 U/mL and 100 μg/mL, respectively), while A549 cells were suspended in Dulbecco’s Modified Eagle Medium (DMEM-F12), containing L-glutamine, 25 mM HEPES, 10% inactivated fetal bovine serum (FBS), and penicillin/streptomycin solution (100 U/mL and 100 μg/mL, respectively) (Basu et al., 2023; Batbold and Liu, 2022).

### 2.10 Cell treatment

PBMC, HL-60, and A549 cells were seeded in a culture medium and incubated with sea buckthorn seed extracts, fractions, or compounds (at the concentrations of 1–100 μg/mL) in 96-well plates at 37 °C and 5% CO_2_. Blood platelets were suspended in Barber’s Buffer and incubated at 37 °C.

### 2.11 Cytotoxicity toward blood platelets (LDH activity assessment)

Lactate dehydrogenase activity was measured with the LDH Cytotoxicity Assay Kit (Item No. 601170, Cayman Chemical). All procedures were carried out according to the kit manual. 200 μL of blood platelets suspended in Barber’s Buffer (2 x 10^7^ platelets/mL) were seeded into 96-well plates. 20 μL of Barber’s Buffer was added to three wells for Background Control, 20 μL of Triton X-100 was added to three wells for Maximum Release, 20 μL of Assay Buffer was added to three wells for Spontaneous Release; 20 μL of sea buckthorn extracts, fractions, or compounds was added to the sample wells in triplicate. The plates were incubated at 37 °C for 1 h. After the incubation, the plates were centrifuged at 400 x g for 5 min. 100 μL of supernatant was transferred to a new 96-well plate, and 100 μL of LDH Reaction Solution was added to the wells. The samples were incubated on an orbital shaker for 30 min at 37 °C. The absorbance was read at 490 nm; cytotoxicity was calculated according to the instruction manual.

### 2.12 Cytotoxicity toward PBMC, HL-60, and A549 cells (resazurin assay)

The viability of PBMC, HL-60, and A549 cells was measured with the resazurin assay, which is based on the ability of living cells to reduce resazurin to resorufin (O’Brien et al., 2000). Resazurin sodium salt powder was dissolved in sterile PBS buffer. 90 μL of the cells was added to 96-well plates at the densities of 50 000 PBMCs/well, 10 000 HL-60 cells/well, or 50 000 A549 cells/well. A549 cells were incubated at 37 °C and 5% CO_2_ for 5 h to allow for cell adhesion. 10 μL of extract vehicle (in the control samples) or sea buckthorn seed extracts, fractions, or compounds were added to the wells. The plates were incubated at 37 °C and 5% CO_2_ for 2 or 24 h. Afterward, 10 μL of resazurin solution was added to all wells, and absorbance was read at 570 nm with a SPECTROstar Nano Microplate Reader (BMG LABTECH, Ortenberg, Germany). The samples were incubated in the same conditions for 2.5–4 h, and the absorbance was read again. Cell viability was calculated as a percentage of the control.

### 2.13 Genotoxic activity (using alkaline comet assay)

The alkaline comet assay was used to assess the genotoxic potential of extracts, fractions, and compounds from raw and roasted sea buckthorn seeds. First, 500 μL of cells (50 000 cells/sample for A549 and HL-50 cells or 100 000 cells/sample for PBMCs) were incubated with the extracts, fractions, and compounds for 2 h at 37 °C. Cells incubated with 10 μM H_2_O_2_ for 15 min on ice were used as a positive control. Each sample was prepared in duplicate. After the incubation, the cells were centrifuged at 1700 rpm for 7 min at room temperature. 450 μL of supernatant was discarded, and the remaining cells were suspended in 0.75% LMP (low melting point) agarose. The cells were spread onto microscope slides precoated with 0.5% NMP (normal melting point) agarose and cooled until the agarose was solid. Afterwards, the slides were incubated for 1 h (at 4 °C) in a lysis buffer (2.5 M NaCl, 0.1 M EDTA, 10 mM TRIS, and 1% Triton X-100, pH 10). After lysis, the slides were washed and immersed in an ice-cold DNA-unwinding alkaline buffer (300 mM NaOH, 1 mM EDTA, pH > 13) for 20 min. Afterwards, the slides were washed with an electrophoresis buffer (30 mM NaOH and 1 mM EDTA), put into an electrophoresis chamber, and submerged in electrophoresis buffer. Electrophoresis was performed for 20 min at 17 V and 32 mA. When the electrophoresis ended, the slides were washed with distilled water and dried overnight. The next day, the samples were stained with 2 μg/mL DAPI for 1 h and analyzed with a fluorescence microscope (Eclipse, Nikon, Tokyo, Japan). Fluorescent imaging was conducted at ×200 magnification; the microscope was equipped with a ProgRes MF cool monochrome camera (JENOPTIK, Jena, Germany). The camera feed was analyzed with a Lucia Comet Assay 7.30 image analysis system (Laboratory Imaging, Prague, Czech Republic). 50 comets were randomly selected from each sample and analyzed; the % of tail DNA was quantified and used as an indicator of DNA damage (Singh et al., 1988; Tokarz et al., 2016).

### 2.14 Apoptosis

Apoptosis was measured with ApoTox-Glo Triplex Assay (Promega, Madison, Wisconsin, USA). This assay uses three fluorescent markers to measure viability, cytotoxicity, and apoptosis of cells. Apoptosis is measured with a luminogenic DEVD-peptide substrate for caspase-3/7 and Ultra-Glo recombinant Thermostable Luciferase. Caspase-3/7 cleavage of the substrate releases luciferin, which is then transformed into luciferase that produces a light signal. First, PBMC, HL-60, and A549 cells were seeded into a black 96-well plate at the densities of 50 000, 10 000, and 10 000 and cells/well, respectively. A549 cells were incubated at 37 °C and 5% CO_2_ for 5 h to allow for cell adhesion. The final volume in all wells was 100 μL. A blank sample consisted of medium and compound vehicle. 5 μM camptothecin was used as a positive control. After the addition of extracts, fractions, or compounds, the cells were incubated for 24 h at 37 °C and 5% CO_2_. Afterward, 20 μL of the viability/cytotoxicity reagent was added to all wells according to the kit manual. The samples were incubated for 30 min at 37 °C, and fluorescence was measured at 400 _Ex_/505 _Em_ (for viability assessment) and 485 _Ex_/520 _Em_ (for cytotoxicity assessment) with a VANTAstar multi-mode microplate reader (BMG LABTECH, Ortenberg, Germany). Next, 100 μL of the Caspase-Glo 3/7 reagent was added to all wells, and the samples were incubated at room temperature for 30 min. Luminescence was measured with a VANTAstar multi-mode microplate reader (BMG LABTECH, Ortenberg, Germany).

### 2.15 Detection of reactive oxygen species

The detection of intracellular reactive oxygen species was carried out with H_2_DCFDA (2,7-dichlorofluorescein diacetate), a cell-permeable reduced form of fluorescein. Upon entering the cell, H_2_DCFDA is de-esterified and oxidized by reactive oxygen species (ROS) into highly fluorescent 2′,7′-dichlorofluorescein (DCF). PBMC and HL-60 cells were washed twice with Hank’s balanced salt solution (HBSS) containing Ca^2+^ and Mg^2+^. H_2_DCFDA in PBS at a concentration of 20 µM was added, and the cells were incubated at 37 °C in the dark for 30 min. The cells were washed twice with HBSS and seeded into 96-well plates at a density of 400 000 (PBMC) or 50 000 (HL-60) cells/well at a total volume of 100 μL. A549 cells were first seeded into 96-well plates at a density of 25 000 cells/well and incubated for 24 h to allow for cell adhesion. Afterward, the cells were carefully washed with HBSS three times, incubated with 20 μM H_2_DCFDA for 30 min in the dark at 37 °C, and washed three times. The extracts, fractions, and compounds were added to the wells at the concentrations of 1 and 50 μg/mL; the compound vehicle was added to the negative control, and 5 μM H_2_O_2_ was added to the positive control. The fluorescence intensity was measured after 0, 15, 30, 60, and 120 min with a VANTAstar multi-mode microplate reader (BMG LABTECH, Ortenberg, Germany) at 495 _Ex_/530 _Em_. Baseline fluorescence (t_0_) was subtracted from the values obtained at 15, 30, 60, and 120 min for each well.

### 2.16 Statistical analysis

Statistical analysis was carried out with Statistica 10 (StatSoft 13.3, TIBCO Software Inc., Palo Alto, CA, USA). Shapiro-Wilk’s test was used to check the data distribution; Levene’s test was used to determine the homogeneity of variance. If the data had a normal distribution and homogenous variance, one-way ANOVA with Tukey’s *post hoc* test was used to assess the differences between groups; otherwise, the Kruskal-Wallis test was used. The results are presented as means ± SD or medians and interquartile ranges. The differences between groups were considered to be significant when p < 0.05. Cohen’s d (*d*) and Confidence Intervals (CI) were calculated for the samples that showed significant differences. Dixon’s test was used to eliminate uncertain data. Exclusion rates for the following assays were: LDH assay – 0.6%; resazurin assay – 0.1%; comet assay – 0%; apoptosis – 0.3%; ROS assay – 0.2%.

## 3 Results

### 3.1 Viability of blood platelets (based on LDH activity)

**FIGURE 1:**
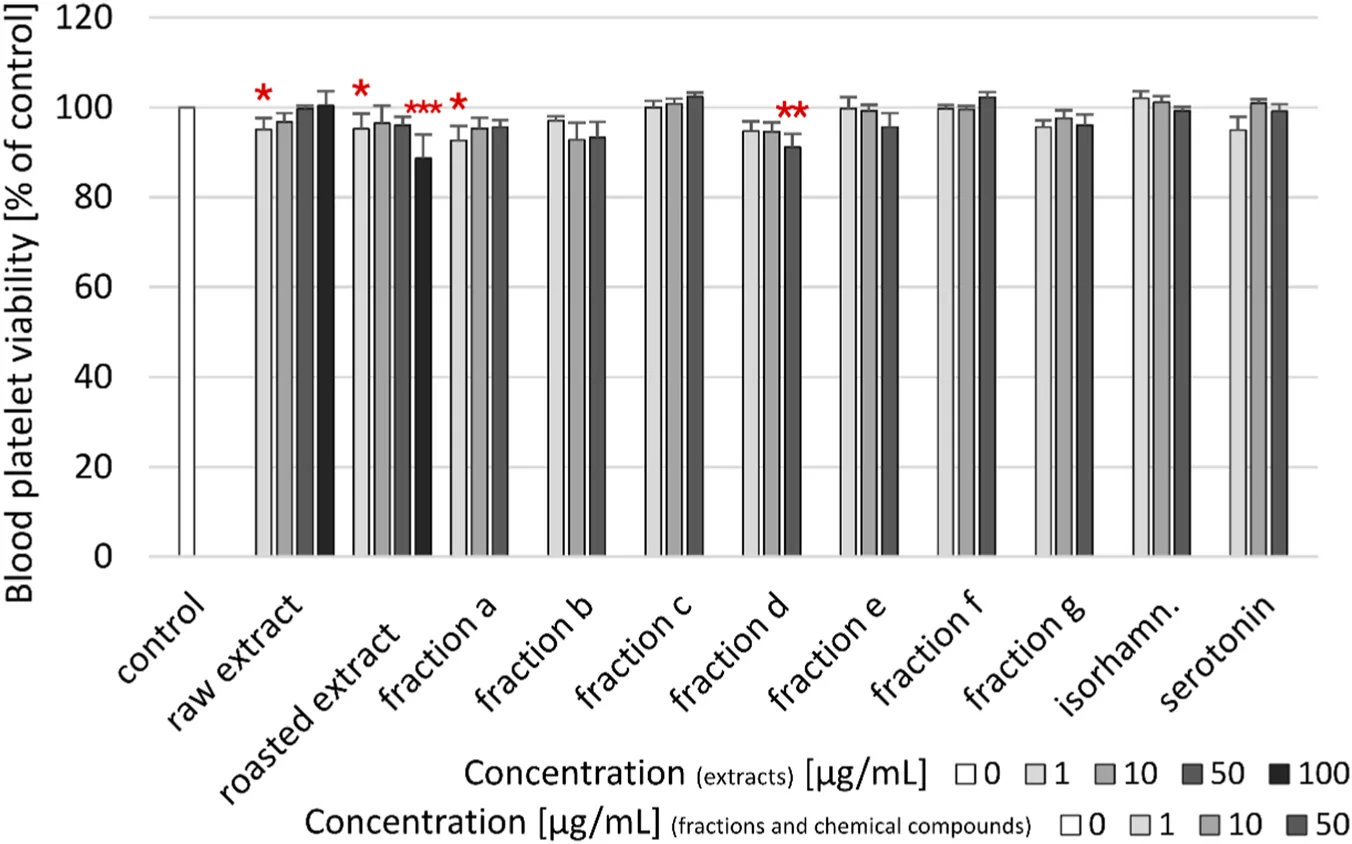
The viability of blood platelets (based on LDH activity) treated with extracts (1–100 μg/mL), fractions (1–50 μg/mL), and chemical compounds (1–50 μg/mL) from sea buckthorn seeds (*n* = 9). The samples were incubated with the extracts, fractions, and compounds for 1 h at 37 °C. The results are expressed as the % of control. Data represent means ± SD. The results were considered significant at p < 0.05 (*p < 0.05, **p < 0.01, ***p < 0.001). Isorhamn. - isorhamnetin 3-*O*-β-glucoside-7-*O*-α-rhamnoside.

The analysis of LDH activity revealed that the extract from roasted sea buckthorn seeds at the concentration of 100 μg/mL decreased blood platelet viability to approximately 88.6% ± 5.3% of control (p < 0.001; 95% CI, 84.6%–92.7%), while fraction d at the concentration of 50 μg/mL decreased it to approximately 91.1% ± 3.0% (p < 0.01; 95% CI, 88.8%–93.4%). Moreover, 1 μg/mL of extracts from raw and roasted seeds and fraction a caused a very slight reduction of viability (p < 0.05). All the remaining fractions, isorhamnetin 3-*O*-β-glucoside-7-*O*-α-rhamnoside, and serotonin did not decrease the viability of blood platelets (Figure 1).

### 3.2 Viability of PBMCs, HL-60, and A549 cells (based on resazurin assay)

**FIGURE 2:**
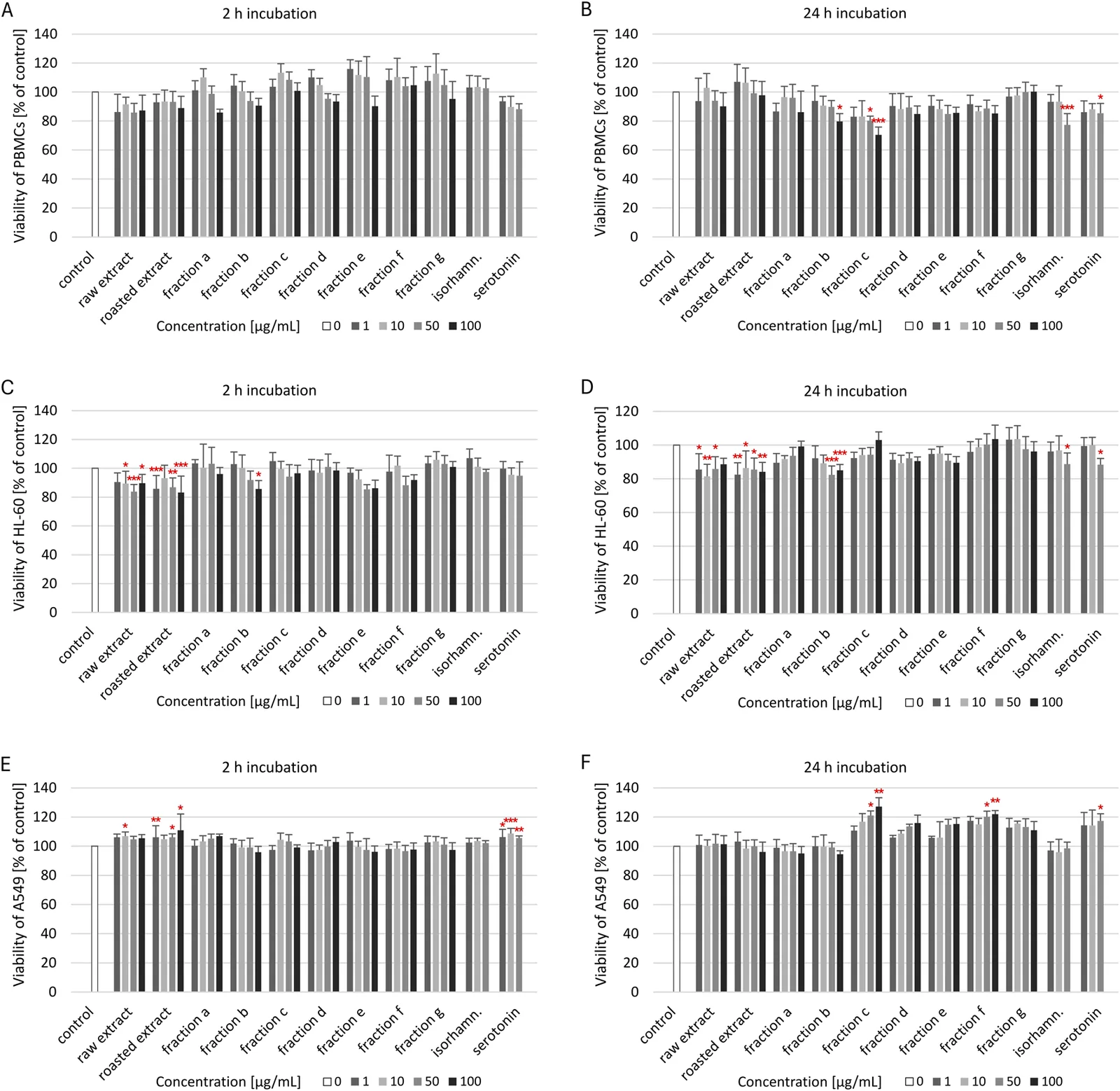
The viability of PBMC, HL-60, and A549 cells (based on resazurin assay), incubated with extracts (1, 10, 50, and 100 μg/mL), fractions (1, 50, and 100 μg/mL), and chemical compounds (1, 10, and 50 μg/mL) from sea buckthorn seeds (*n* = 6–12). **(A)** PBMC, 2 h incubation; **(B)** PBMC, 24 h incubation; **(C)** HL-60, 2 h incubation; **(D)** HL-60, 24 h incubation; **(E)** A549, 2 h incubation; **(F)** A549, 24 h incubation. The results are expressed as the % of control. Data represent means ± SD. The results were considered significant at p < 0.05 (*p < 0.05, **p < 0.01, ***p < 0.001). Isorhamn. - isorhamnetin 3-*O*-β-glucoside-7-*O*-α-rhamnoside.

None of the tested sea buckthorn extracts, fractions or compounds affected the viability of PBMC cells when the incubation time was 2 h (Figure 2A). However, 24 h incubation with fraction b (at the concentration of 100 μg/mL), fraction c (50 and 100 μg/mL), isorhamnetin 3-*O-*β-glucoside-7-*O*-α-rhamnoside (50 μg/mL), and serotonin (50 μg/mL) significantly reduced the level of resazurin conversion to resorufin by 14.7%–29.6%. The strongest effect was observed for fraction c–resazurin reduction in cells incubated with this fraction at 50 μg/mL was decreased to 80.3% (p < 0.05; 95% CI, 77.1%–83.5%), while 100 μg/mL decreased it to 70.4% (p < 0.001; 95% CI, 64.7%–76.1%). Moreover, incubation of PBMC cells with 50 μg/mL isorhamnetin 3-*O-*β-glucoside-7-*O*-α-rhamnoside inhibited resazurin conversion by 22.6% (p < 0.001; 95% CI, 69.2%–85.5%) (Figure 2B).

Fraction c did not affect the viability of HL-60 cells; however, fraction b showed a cytotoxic effect toward this cancer cell line at both incubation times. Fraction b (at the concentration of 100 μg/mL), raw sea buckthorn seeds extract (at 10–100 μg/mL), and roasted seeds extract (at 1, 50, and 100 μg/mL) significantly lowered the levels of resazurin reduction by HL-60 cells by 10.3%–16.8% after 2 h of incubation (Figure 2C). 24 h incubation of HL-60 cells with raw seed extract (at 1–50 μg/mL) and roasted seeds extract (at 1–100 μg/mL) significantly decreased resazurin reduction by 13.7%–18.5%; for example, resazurin conversion was reduced to 85.8% by 50 μg/mL raw extract (p < 0.05; 95% CI, 78.2%–93.44%), to 85.5% by 50 μg/mL roasted extract (p < 0.05; 95% CI, 78.4%–92.6%), and to 84.2% by 100 μg/mL roasted extract (p < 0.01; 95% CI, 78.3%–90.1%). 50 μg/mL fraction b decreased resazurin reduction to 82.4% (p < 0.001; 95% CI, 79.1%–85.7%) and 100 μg/mL fraction b to 85.0% (p < 0.001; 95% CI, 82.8%–87.2%); 50 μg/mL isorhamnetin 3-*O*-β-glucoside-7-*O*-α-rhamnoside reduced resazurin conversion to 88.7% (p < 0.05; 95% CI, 81.9%–95.6%), and 50 μg/mL serotonin to 88.4% (p < 0.05; 95% CI, 84.4%–92.3%) (Figure 2D).

On the other hand, the conversion of resazurin to resorufin by A549 cells was enhanced in some of the samples, indicating higher viability or metabolic activity. Serotonin increased the levels of resazurin conversion in A549 cells after both 2 h (significant effect at 1–50 μg/mL, 5.6%–8.7% increase) and 24 h (significant effect at 50 μg/mL, viability increased by 17.3% (p < 0.05; 95% CI, 114.2%–120.4%) (Figures 2E,F). After 24 h incubation, 50 μg/mL fractions c and f (both rich in serotonin) increased resazurin conversion to resorufin by 21.1% (p < 0.05; 95% CI, 117.9%–124.3%) and 20.1% (p < 0.05; 95% CI, 116.0%–124.2%), respectively. 100 μg/mL fraction c increased resazurin reduction by 27.2% (p < 0.01; 95% CI, 120.9%–133.5%), while 100 μg/mL fraction f increased it by 22.0% (p < 0.01; 95% CI, 119.5%–124.5%) (Figure 2F). Interestingly, 10 μg/mL of raw seed extract and 1, 50, and 100 μg/mL of roasted seeds extract significantly enhanced the level of resazurin reduction by A549 cells only after 2 h incubation, though this effect was small (6.1%–10.9%) (Figure 2E).

### 3.3 Genotoxic activity in PBMCs, HL-60, and A549 cells (measured with alkaline comet assay)

**FIGURE 3:**
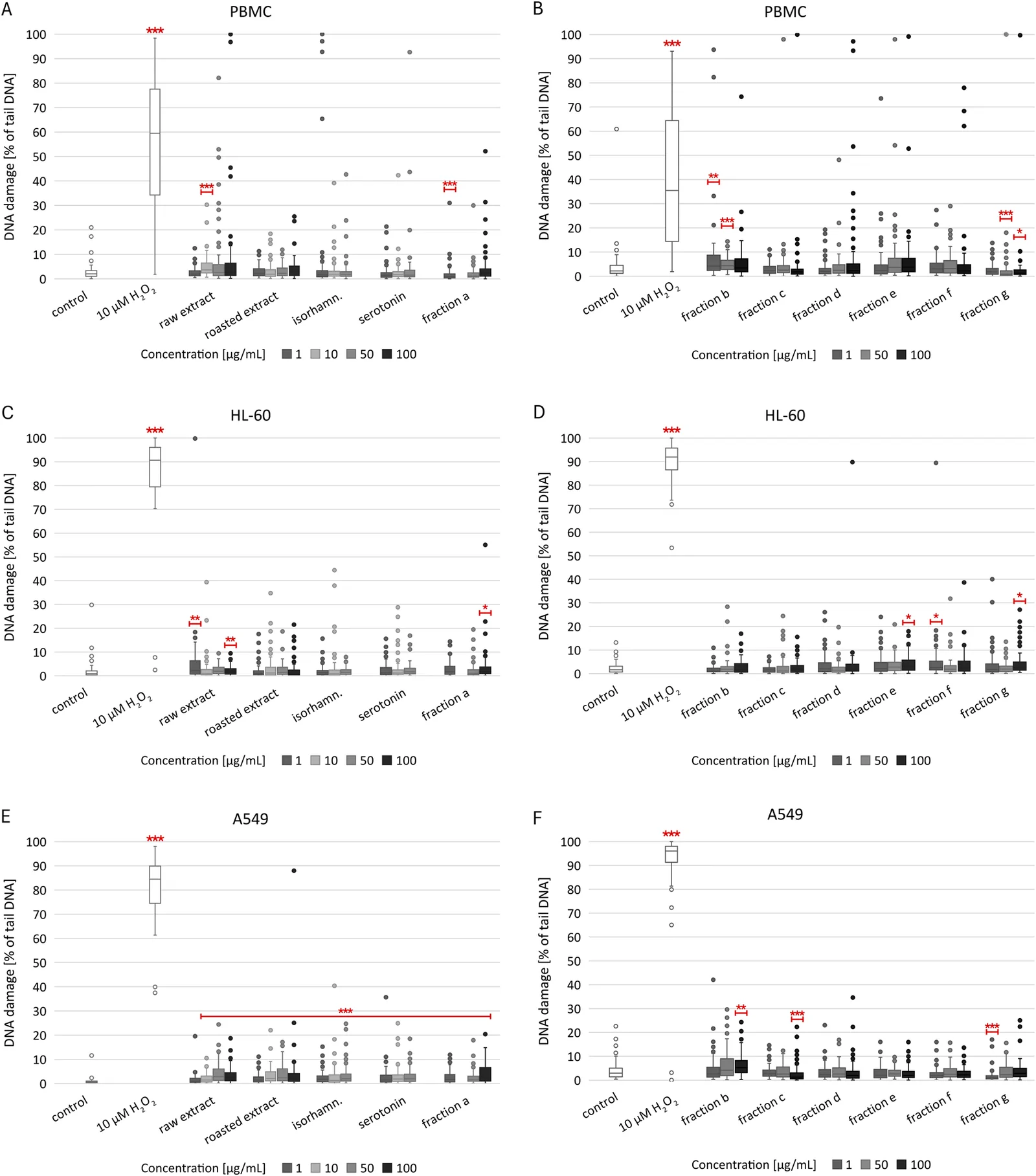
DNA damage (expressed as the % of comet tail DNA) of PBMC **(A,B)**, HL-60 **(C,D)**, and A549 cells **(E,F)** incubated for 2 h with extracts (1, 10, 50, and 100 μg/mL), fractions (1, 50, and 100 μg/mL), and chemical compounds (1, 10, and 50 μg/mL) from sea buckthorn seeds (*n* = 100). 10 μM H_2_O_2_ was used as a positive control. Data are presented as medians and interquartile ranges. The results were considered significant at p < 0.05 (*p < 0.05, **p < 0.01, ***p < 0.001). Isorhamn. - isorhamnetin 3-*O*-β-glucoside-7-*O*-α-rhamnoside.

2 h incubation of raw sea buckthorn seed extract (10 μg/mL) with PBMC cells significantly increased their DNA damage measured by the alkaline comet assay from 2.76% to 5.09% (p < 0.001; *d* = 0.59; 95% CI, 4.2%–6.0%). Fraction b increased DNA damage from 3.58% to 7.29% (1 μg/mL; p < 0.01; *d* = 0.37; 95% CI, 4.8%–9.8%) and to 4.92% (50 μg/mL; p < 0.001; *d* = 0.27; 95% CI, 4.3%–5.5%). Fraction a (1 μg/mL) decreased the percentage of tail DNA of PBMCs from 2.76% to 1.77% (p < 0.001; *d* = 0.3; 95% CI, 1.1%–2.5%), 50 μg/mL fraction g decreased it from 3.58% to 2.86% (p < 0.001; *d* = 0.09; 95% CI, 0.8%–4.9%), and 100 μg/mL fraction g to 2.84% (p < 0.05; *d* = 0.09; 95% CI, 0.86%–4.8%) (Figures 3A,B).

Raw seed extract (1 and 50 μg/mL), fraction a (100 μg/mL), fraction e (100 μg/mL), fraction f (1 μg/mL), and fraction g (100 μg/mL) had a small genotoxic effect on HL-60 cells – DNA damage was increased by 0.75–1.33 percentage points (Figures 3C,D).

2 h incubation of A549 cells with raw seed extract (10–100 μg/mL), roasted seeds extract (1–100 μg/mL), fraction a (1–100 μg/mL), fraction b (100 μg/mL), isorhamnetin 3-*O*-β-glucoside-7-*O*-α-rhamnoside (1–50 μg/mL), and serotonin (1–50 μg/mL) caused significant DNA damage, however, the changes in tail DNA were small–they ranged between 0.64 and 2.23 percentage points. Additionally, fraction c (100 μg/mL) and fraction g (1 μg/mL) decreased DNA damage by 1.67 (p < 0.001; *d* = 0.37; 95% CI, 1.9%–3.3%) and 1.82 (p < 0.001; *d* = 0.69; 95% CI, 1.3%–2.2%) percentage points, respectively (Figures 3E,F).

### 3.4 Apoptosis (based on caspase 3/7 activity)

**FIGURE 4:**
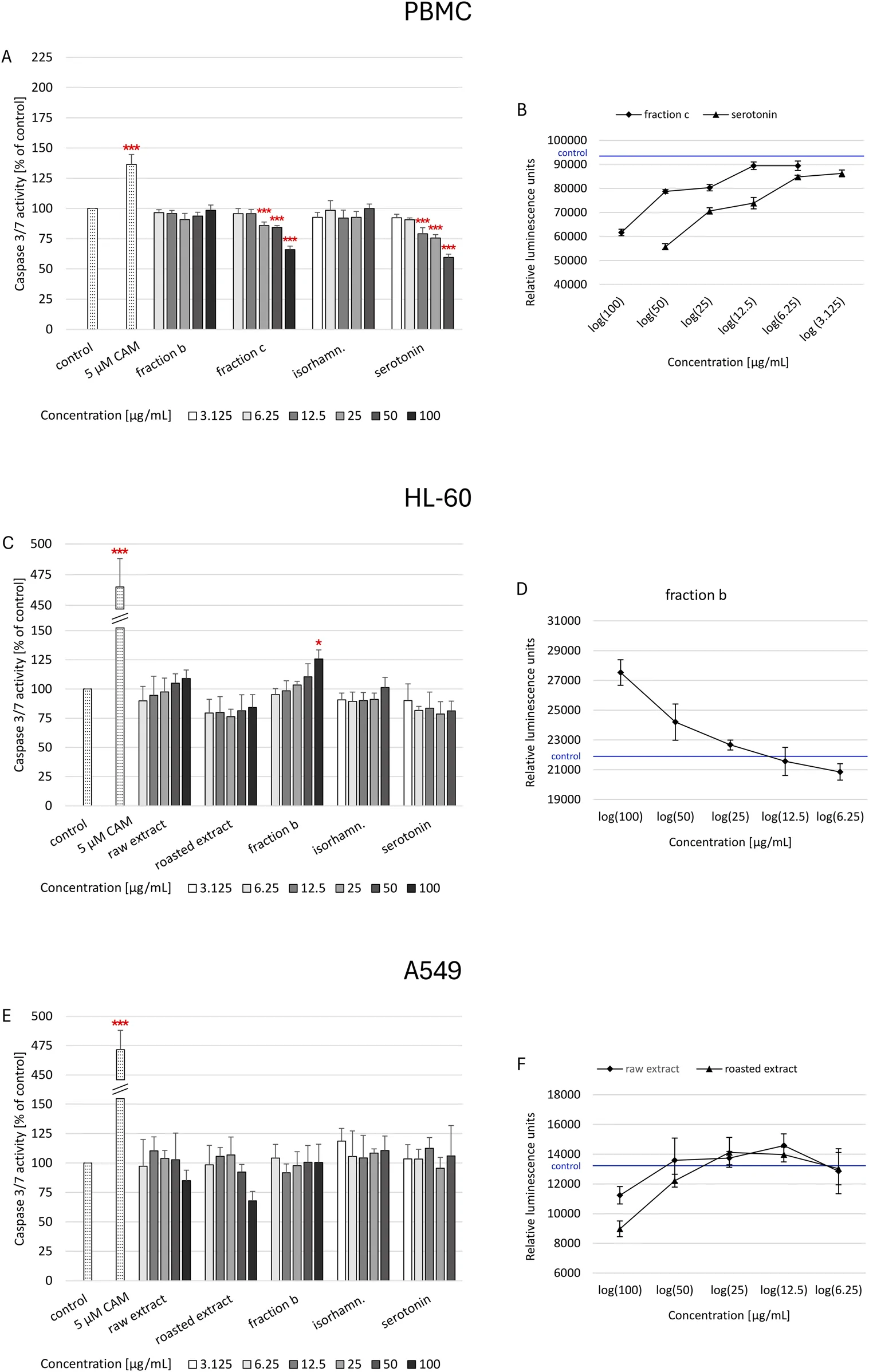
The % of caspase 3/7 activity (a marker of apoptosis) in PBMC **(A)**, HL-60 **(C)**, and A549 **(E)** cells after 24 h incubation relative to control (*n* = 4). 5 μM camptothecin (CAM) was used as a positive control. Data are presented as means ± SD. The results were considered significant at p < 0.05 (*p < 0.05, ***p < 0.001). Graphs **(B,D,F)** show relative luminescence units plotted against log-transformed mean values of the control samples. Isorhamn. - isorhamnetin 3-*O*-β-glucoside-7-*O*-α-rhamnoside.

An assay based on caspase-3/7 activity was used to assess the ability of selected preparations from sea buckthorn seeds to induce apoptosis. Both fraction c (containing serotonin) and serotonin (25–100 μg/mL) significantly decreased the level of caspase 3/7 in comparison to the control in PBMC cells after 24 h of incubation, indicating a reduced level of apoptosis in the wells (Figures 4A,B). Fraction b (100 μg/mL) significantly induced apoptosis in HL-60 cells; caspase 3/7 level was increased by approximately 25% (p < 0.01; *d* = 4.22; 95% CI, 113.2%–138.1%). However, no such effect was observed for both extracts, isorhamnetin 3-*O*-β-glucoside-7-*O*-α-rhamnoside, or serotonin (Figures 4C,D). In A549 cells, the level of caspase 3/7 was decreased by extracts from raw and roasted seeds at 100 μg/mL, but this effect was not statistically significant (Figures 4E,F).

**FIGURE 5:**
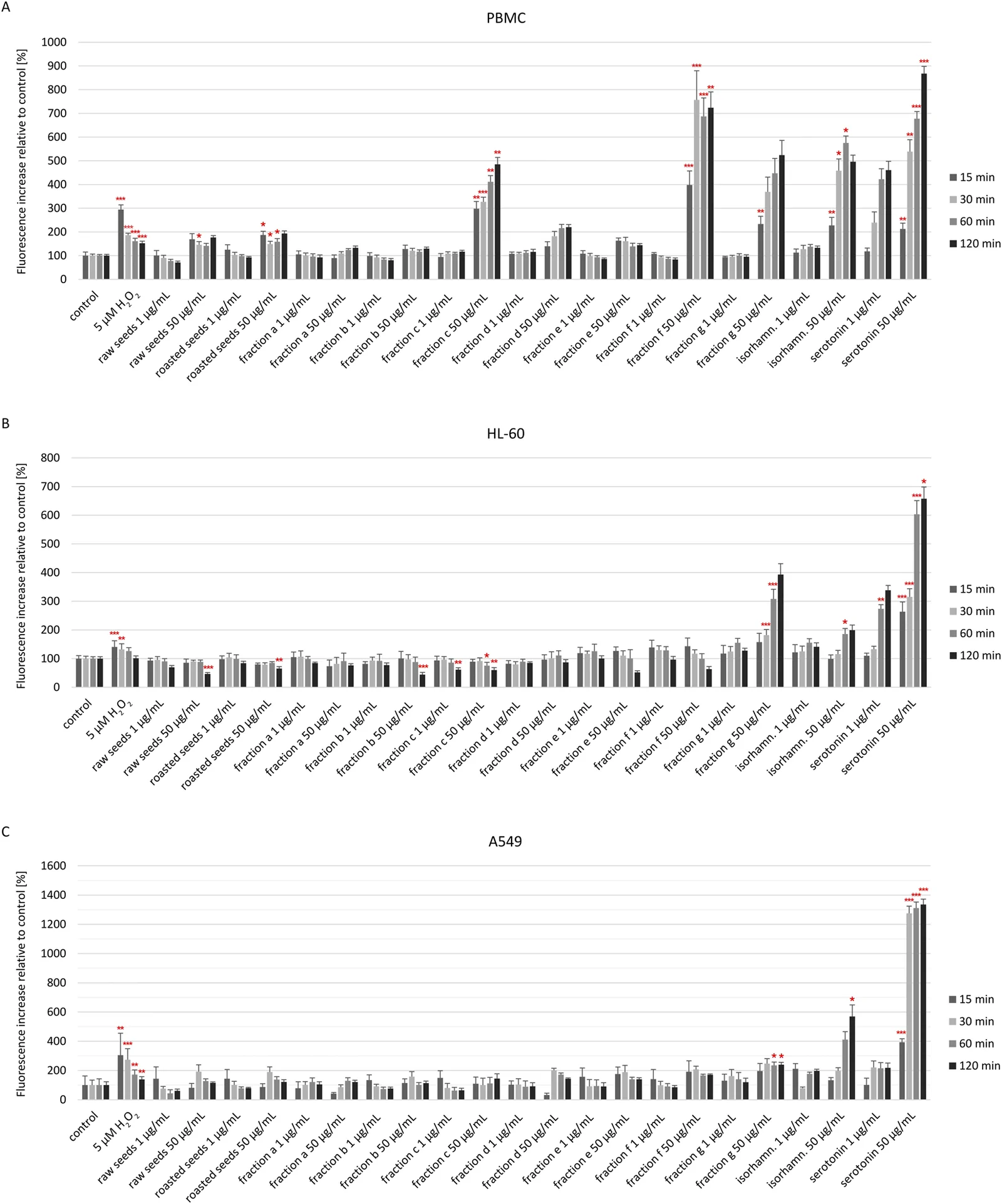
ROS generation (based on DCF fluorescence) in PBMC **(A)**, HL-60 **(B)**, and A549 **(C)** cells incubated with extracts, fractions, and chemical compounds from sea buckthorn seeds, measured at 15, 30, 60, and 120 min of incubation (*n* = 7). 5 μM H_2_O_2_ was used as a positive control. Data are presented as means ± SD. Baseline relative fluorescence intensity values were subtracted from the values obtained after 15, 30, 60, and 120 min of incubation. The fluorescence increase in the tested samples was then expressed as the percentage of the increase observed in the control sample. The results were considered significant at p < 0.05 (*p < 0.05, **p < 0.01, ***p < 0.001). Isorhamn. - isorhamnetin 3-*O*-β-glucoside-7-*O*-α-rhamnoside.

### 3.5 ROS generation

To measure the generation of ROS in various cells (PBMC, HL-60, and A549 cells), DCF (2′,7′-dichlorofluorescein) fluorescence was monitored 15, 30, 60, and 120 min after adding the extracts, fractions, and chemical compounds from sea buckthorn seeds. 50 μg/mL of raw and roasted seed extracts, fraction c, fraction f, fraction g, isorhamnetin 3-*O*-β-glucoside-7-*O*-α-rhamnoside, and serotonin all significantly increased ROS generation in PBMC cells. This effect was strongest at 50 μg/mL serotonin and fraction f, but fraction c, fraction g, and isorhamnetin 3-O-β-glucoside-7-O-α-rhamnoside also significantly increased ROS production.

In HL-60 cells, ROS generation was decreased by the extracts from raw and roasted seeds and fraction b at 50 μg/mL after 120 min of incubation. Moreover, fraction c at the concentration of 1 μg/mL decreased ROS levels after 120 min of incubation, while at 50 μg/mL, the effect was significant after 60 and 120 min. On the other hand, fraction g, isorhamnetin 3-*O*-β-glucoside-7-*O*-α-rhamnoside, and serotonin significantly increased ROS generation in HL-60 cells. The strongest effect was observed in fraction g at 50 μg/mL and serotonin at 1 and 50 μg/mL, while 3-*O*-β-glucoside-7-*O*-α-rhamnoside produced a more moderate response.

A similar result was observed in A549 cells; however, here, only a high concentration of serotonin (50 μg/mL) had a significant stimulating effect on ROS generation. Isorhamnetin 3-*O*-β-glucoside-7-*O*-α-rhamnoside and fraction g (at 50 μg/mL) also increased ROS generation; isorhamnetin 3-*O*-β-glucoside-7-*O*-α-rhamnoside produced a moderate response, while fraction g increased ROS levels only to a small degree. However, the remaining fractions and extracts did not significantly affect ROS production in A549 cells.

Table 1 demonstrates a comparison of effects of two extracts (from raw and roasted seeds), seven fractions (three fractions (a, b, and c) from raw sea buckthorn seeds and four fractions (d, e, f, and g) from roasted sea buckthorn seeds), and two chemical compounds: isorhamnetin 3-*O*-β-glucoside-7-*O*-α-rhamnoside, and serotonin (50 μg/mL, the highest concentration that could realistically be achieved in blood through oral supplementation with phenolic compounds) on viability of cells, DNA damage, apoptosis, and ROS production.

**TABLE 1:** A comparison of effects of two extracts (from raw and roasted seeds), seven fractions (three fractions (a, b, and c) from raw sea buckthorn seeds and four fractions (d, e, f, and g) from roasted sea buckthorn seeds), and two chemical compounds: isorhamnetin 3-*O*-β-glucoside7-*O*-α-rhamnoside, and serotonin (50 μg/mL) on viability of cells, oxidative damages of DNA, apoptosis, and ROS production. Numbers in brackets indicate the approximate % of inhibition/effect increase compared to control.

Cells and incubation times	Extract from raw seeds [50 μg/mL]	Extract from roasted seeds [50 μg/mL]	Fraction a [50 μg/mL]	Fraction b [50 μg/mL]	Fraction c [50 μg/mL]	Fraction d [50 μg/mL]	Fraction e [50 μg/mL]	Fraction f [50 μg/mL]	Fraction g [50 μg/mL]	Isorhamnetin 3-*O*-β-glucoside-7-*O*-α-rhamnoside [50 μg/mL]	Serotonin [50 μg/mL]
Viability [% of viability changes relative to control]											
Blood platelets (1 h incubation)	ns	ns	ns	ns	ns	Cytotoxic effect (8.9%)	ns	ns	ns	ns	ns
PBMCs (2 h incubation)	ns	ns	ns	ns	ns	ns	ns	ns	ns	ns	ns
PBMCs (24 h incubation)	ns	ns	ns	ns	Cytotoxic effect (19.7%)	ns	ns	ns	ns	Cytotoxic effect (22.6%)	Cytotoxic effect (14.7%)
HL-60 cells (2 h incubation)	Cytotoxic effect (16.1%)	Cytotoxic effect (13.2%)	ns	ns	ns	ns	ns	ns	ns	ns	ns
HL-60 cells (24 h incubation)	Cytotoxic effect (14.2%)	Cytotoxic effect (14.5%)	ns	Cytotoxic effect (17.6%)	ns	ns	ns	ns	ns	Cytotoxic effect (11.3%)	Cytotoxic effect (11.6%)
A549 cells (2 h incubation)	ns	Increased viability (6.1%)	ns	ns	ns	ns	ns	ns	ns	ns	Increased viability (5.6%)
A549 cells (24 h incubation)	ns	ns	ns	ns	Increased viability (21.1%)	ns	ns	Increased viability (20.1%)	ns	ns	Increased viability (17.3%)
DNA damage [changes in tail DNA relative to control in percentage points]											
PBMCs (2 h incubation)	ns	ns	ns	Genotoxic effect (2.2%)	ns	ns	ns	ns	Decreased damage (1.34%)	ns	ns
HL-60 cells (2 h incubation)	Genotoxic effect (1.1%)	ns	ns	ns	ns	ns	ns	ns	ns	ns	ns
A549 cells (2 h incubation)	Genotoxic effect (2.2%)	Genotoxic effect (1.7%)	Genotoxic effect (0.9%)	ns	ns	ns	ns	ns	ns	Genotoxic effect (1.4%)	Genotoxic effect (1.4%)
Apoptosis											
PBMCs (24 h incubation)	-	-	-	ns	Decreased apoptosis (15.7%)	-	-	-	-	ns	Decreased apoptosis (40.4%)
HL-60 cells (24 h incubation)	ns	ns	-	ns	-	-	-	-	-	ns	ns
A549 cells (24 h incubation)	ns	ns	-	ns	-	-	-	-	-	ns	ns
ROS production											
PBMCs (2 h incubation)	Increased ROS	Increased ROS	ns	ns	Increased ROS	ns	ns	Increased ROS	Increased ROS	Increased ROS	Increased ROS
HL-60 cells (2 h incubation)	Decreased ROS	Decreased ROS	ns	Decreased ROS	Decreased ROS	ns	ns	ns	Increased ROS	Increased ROS	Increased ROS
A549 cells (2 h incubation)	ns	ns	ns	ns	ns	ns	ns	ns	Increased ROS	Increased ROS	Increased ROS

“ns” – not significant; “-” – effect not studied.

## 4 Discussion

Several authors studied the toxicity of different sea buckthorn organs, both *in vitro* and in animal models. For example, Martins de Deus et al. (2023) reported that the GI_50_ values (concentration leading to 50% inhibition of proliferation) of sea buckthorn branches, twigs, leaves and flowers hydroethanolic extract were 183–209 μg/mL in tumoral AGS (gastric adenocarcinoma), CaCo-2 (colorectal adenocarcinoma), and MCF-7 (breast adenocarcinoma) cells, 219 μg/mL in non-tumoral VERO (African green monkey kidney) cells, and 178 μg/mL in PLP_2_ (pig liver primary culture) cells. The fact that the GI_50_ value was lower in PLP_2_ than in tumor cells suggests that this extract would not be suitable for the treatment of these cancer types. The authors also studied the *in vivo* acute toxicity of the extract toward *Artemia franciscana.* The LC_50_ (concentration lethal to 50% of individuals) value was under 400 mg/L, which suggests low toxicity (Martins de Deus et al., 2023). Furthermore, Gore et al. (2024) determined that a nanoemulsion from *H. rhamnoides* fruit oil was not toxic to female rats after 14 days of dermal administration (Gore et al., 2024). Marciniak et al. (2021) showed that low-polarity fractions from sea buckthorn fruits, leaves, and twigs have cytotoxic effects toward HT29 (colorectal adenocarcinoma), HCT116 (colorectal carcinoma), PC3 (prostate adenocarcinoma), MCF-7, and AGS cancer cell lines (IC_50_ values from 14.6 to 40.4 μg/mL). The effect of the fractions on PC3 and HT29 was the strongest out of all the cell lines (IC_50_ from 14.6 to 27.1 μg/mL). Notably, IC_50_ values in normal human cells (HS27 (foreskin fibroblasts) and PBMC) were much higher (37.1–74.6 μg/mL), suggesting that low concentrations of low-polarity sea buckthorn extracts are good candidates for anticancer therapy (Marciniak et al., 2021). In another study, saponified sea buckthorn berry extract rich in carotenoids exhibited anti-tumor activity in two breast cancer cell lines–BT-549 (IC_50_ = 22.6 μg/mL) and T47D (IC_50_ = 34.9 μg/mL). Cell death induced by the extract occurred mainly by apoptosis (Visan et al., 2023). Dvorska et al. (2025) also reported that sea buckthorn peel extracts were cytotoxic toward breast cancer cells. They inhibited the growth of MCF-7 and MDA-MB-231 (breast adenocarcinoma) cell lines. The effectiveness of sea buckthorn fruit peels against breast cancer was also studied *in vivo*. 3 and 30 g/kg of powdered fruit peels were fed to rats for 1 week before carcinogen induction; this treatment was continued for 14 weeks. Sea buckthorn supplementation was well-tolerated and did not cause any macroscopic organ abnormalities or other side effects. The lower dose was equivalent to human consumption of 20 g of dried fruit per day. Both doses significantly reduced tumor volume (by 43%–48%) and mitotic index of cancer cells (by 34%–44.5%). Sea buckthorn treatment increased the expression of two apoptotic markers–Bcl-2-associated X protein (Bax) and caspase-3. Moreover, the expression of three tumor-suppressive miRNAs (miR-10a-5p, miR-322-5p, and miR-450a-5p) was upregulated in breast cancer tissue (Dvorska et al., 2025). In another study, a hydroalcoholic extract of sea buckthorn seed residues decreased the viability of B16F10 (mouse melanoma) cells. The effect was the strongest after 72 h treatment (IC_50_ = 155.38 μg/mL), but much weaker after 24 h incubation (IC_50_ = 524.78 μg/mL) (Zhang et al., 2018). Stochmal et al. (2021) tested the effect of fractions from sea buckthorn leaves and twigs on a non-tumorous human fibroblast line (HFF-1). After 24 h incubation, fractions either did not affect the fibroblasts at all or did so only at high concentrations (above 100 μg/mL) (Stochmal et al., 2021).

In our previous study, Juszczak et al. (2021) observed that a saponin fraction from sea buckthorn leaves stimulated the growth of HL-60 cells at lower concentrations (0.5 and 1 μg/mL), but decreased their viability at 50 and 100 μg/mL. 24 h incubation with 100 μg/mL of sea buckthorn saponins resulted in approximately 73% viability in comparison to the control (Juszczak et al., 2021).

Our study is the first work devoted to a comprehensive assessment of toxicity of two extracts (from raw and roasted seeds), seven fractions (three fractions (a, b, and c) from raw sea buckthorn seeds and four fractions (d, e, f, and g) from roasted sea buckthorn seeds), and two chemical compounds (isorhamnetin 3-*O*-β-glucoside-7-*O*-α-rhamnoside and serotonin), employing several *in vitro* assays on healthy (human blood platelets, and PBMC) and cancer (HL-60, and A549) cells. Their cytotoxicity and genotoxicity were examined in the concentration range 1–100 μg/mL, which was chosen based on our previous studies and literature data. Manach et al. (2004) indicate that the achievable concentrations of phytochemicals in blood are mostly at levels ranging from nanomoles to a few micromoles per liter (Manach et al., 2004). The toxicity of the extracts, fractions, serotonin, and isorhamnetin 3-*O*-β-glucoside-7-*O*-α-rhamnoside in PBMC cells, HL-60, and A549 cells was estimated after 2 h and 24 h incubation, using the resazurin-based metabolic assay. Based on the results of our cytotoxicity tests, the viability of HL-60 cells was decreased by the extracts from both raw and roasted sea buckthorn seeds by a similar degree, suggesting that thermal processing does not affect their cytotoxic activity. Fraction b (eluted with 66% methanol from raw seed extract; containing different flavonol glycosides, including isorhamnetin 3-*O*-glucoside-7-*O*-rhamnoside, whose peak constituted about 4.3% of the total CAD peak area) also decreased the viability of HL-60 cells. Isorhamnetin 3-*O*-glucoside-7-*O*-rhamnoside itself also showed a similar effect, suggesting that it likely contributes to the activity of fraction b. Fraction e, which is analogous to fraction b (it is also eluted with 66% methanol), but is derived from roasted seed extract, slightly decreased the viability of HL-60 cells as well, but this effect was not statistically significant. This indicates that thermal processing might have degraded the flavonoids responsible for the anticancer activity of fraction b toward HL-60 cells, which would explain why fraction e had no significant activity. In our previous study, phytochemical analysis revealed that thermal processing decreased the content of flavonoids in sea buckthorn seeds by about 30% (Sławińska et al., 2023). Moreover, CAD peaks of acylated flavonoids in fraction e were generally lower than those of fraction b (Sławińska et al., 2024b). Thermal processing can cause different chemical modifications to polyphenols, including hydrolyzation, oxidation, or dimerization (Xiao, 2022). Heating can lower the content of phenolic acids through thermal degradation, polymerization into insoluble compounds, or volatilization (Liu et al., 2024b). High temperatures can also break down glycoside bonds. For example, the concentration of quercetin glycosides decreased with longer roasting times, while the level of quercetin aglycones increased (Górecki and Hallmann, 2020). It is also important to consider that flavonoid glycosides can also be converted into their aglycone forms by glucosidases, influencing their biochemical and biophysical properties, as well as biological activity (Slámová et al., 2018). For example, quercetin glucosides can be deglycosylated by Caco-2 cell’s intracellular β-glucosidase (Boyer et al., 2005). However, the significance of this process in PBMC, HL-60, and A549 cells is unknown. Some β-glucosidase activity can also be present in FBS used in cell culture, even after heat inactivation (Theron et al., 1994).

The anticancer activities of both fraction b and isorhamnetin 3-*O*-glucoside-7-*O*-rhamnoside were poorer than those observed by Marciniak et al. (2021), who tested the effect of low-polarity fractions from sea buckthorn twigs, leaves, and fruits on the viability of HT29, PC3, AGS, HCT116, and MCF7 cells. Here, after 24 h incubation with 50 μg/mL of fraction b and isorhamnetin 3-*O*-glucoside-7-*O*-rhamnoside resazurin reduction was inhibited by 17.6% and 11.3%, respectively, while low-polarity fractions had IC_50_ values ranging from 14.58 to 40.42 μg/mL. However, it is important to note that in the study by Marciniak et al. (2021), the incubation time was 72 h, which makes it hard to compare these results with our study.

5-HT (serotonin) receptors in HL-60 cells play a modulatory role in key cellular processes such as proliferation, differentiation, and immune-like responses. Activation of different receptor subtypes can influence intracellular signaling pathways, including calcium mobilization, protein kinase C (PKC), and cAMP dependent mechanisms. These pathways are particularly relevant during the differentiation of HL-60 cells into granulocyte or monocyte like phenotypes, where serotonin can affect oxidative burst activity and cytokine production. Although the exact receptor profile is not fully defined, 5-HT_1_ and 5-HT_2_ receptors are most consistently implicated, with possible involvement of other subtypes under specific conditions. Overall, serotonin signaling contributes to the regulation of functional maturation and inflammatory behavior in this leukemia cell model (Sharp and Barnes, 2020; Zhang et al., 2020; Parajulee and Kim, 2023; Cummins et al., 2025). Interestingly, for the first time, we showed that serotonin (50 μg/mL) decreased the reduction of resazurin to resorufin in HL-60 cells as well. The present study also examined the viability of human blood platelets incubated with the preparations from sea buckthorn seeds after 1 h incubation. The raw extract (1 μg/mL), roasted extract (1 and 100 μg/mL), fraction a (1 μg/mL), and fraction d (50 μg/mL) slightly decreased blood platelet viability, but other tested extracts, fractions, serotonin, and isorhamnetin 3-*O*-glucoside-7-*O*-rhamnoside were safe for human blood platelets *in vitro*.

Sea buckthorn pulp oil showed a synergistic anticancer effect against non-small lung cancer cell lines (A549 and H23) when it was paired with Docetaxel. At the lowest dose (0.1 nM) Doceltaxel showed almost no cytotoxicity toward lung cancer cells, but when it was administered together with sea buckthorn oil at the concentration of 1 mg/mL, the viability was decreased by 24%–32%. The oil changed cell morphology–after 24 h incubation, it increased cell size and flattened shape could be observed in both A549 and H23. Subsequent experiments confirmed that it induced senescence in these cell lines (Batbold and Liu, 2022). On the other hand, Podder et al. (2013) report that sea buckthorn leaf extract had a cytoprotective effect on A549 cells treated with paraquat, a harmful herbicide. At a concentration of 200 μg/mL, the extract was not cytotoxic to A549 cells. On the contrary, it protected them from paraquat-induced toxicity by preserving membrane integrity, decreasing ROS generation, and activating Nrf2 (nuclear factor erythroid 2-related factor 2) transcription factor. Moreover, the expression of NAD(P)H dehydrogenase quinone 1 (*NQO1*), glutathione peroxidase 1 (*GPX1*), glutathione reductase (*GSR*), and catalase (*CAT*) antioxidant genes was upregulated by the extract (Podder et al., 2013). Here, the extracts and fractions from sea buckthorn seeds did not inhibit the growth of A549 cells. On the contrary, a slight increase in their viability or metabolic activity could be observed in both raw and roasted extract after 2h incubation. Moreover, serotonin and serotonin-containing fractions c and f enhanced resazurin reduction in A549 cells during 24 h incubation. The CAD peak of serotonin in fraction c was about 28.6% of the total peak area (Sławińska et al., 2024b). Taş et al. (2018) report, that roasting at 150 °C for 30 min did not cause serotonin degradation in four out of five Turkish hazelnut varieties, though its thermal stability might be due to its localization in the food matrix or other protective mechanisms, so the results of thermal processing might vary between different plant species (Taş et al., 2018). On the other hand, procyanidin CAD peaks of fraction c were approximately two times higher than those of fraction f (Sławińska et al., 2024b); this is because procyanidins are easily degraded into (+)-catechin and (-)-epicatechin during thermal processing (Jiang et al., 2023). These results are supported by the findings of Tu et al. (2022), where 25 μM serotonin increased the migrative potential of A549 cells. Furthermore, NSCLC patients with metastasis had increased serotonin content in tumor tissues when compared to non-metastatic patients. The mechanism by which 5-HT promotes cancer progression was tied to the upregulation of the c-Myc signaling pathway through Tribbles pseudokinase 3 (TRIB3). Moreover, c-Myc increased the expression of SLC6A4 (a serotonin transporter), which promoted a 5-HT/c-Myc/SLC6A4/5-HT signaling loop formation (Tu et al., 2022). Apart from serotonin and its derivatives, fractions c and f also contained B-type proanthocyanidins (dimers and trimers of (epi)catechin, and/or (epi)gallocatechin), putative nucleosides (uridine, guanosine, xanthosine), aromatic amino acids (phenylalanine, tryptophan), and methylgallic acid; these compounds might also be responsible for the activity of fractions c and f but require further testing to form definite conclusions (Sławińska et al., 2024b).

Wen et al. (2020) studied the genotoxic and teratogenic potential of sea buckthorn berry oil. None of the tested concentrations (8–5,000 μg/plate) had any mutagenic effects on *Salmonella typhimurium*. Moreover, mice supplemented with 2.34–9.36 g/kg of oil for 5 days didn’t show any signs of toxicity, teratogenicity, or DNA damage (which was assessed by sperm abnormality test and bone marrow micronucleus test) (Wen et al., 2020). In our present experiments, the alkaline version of the comet assay was used to assess the level of DNA damage caused by the tested extracts, fractions, serotonin, and isorhamnetin 3-*O*-β-glucoside-7-*O*-α-rhamnoside. For the first time, our results showed that raw sea buckthorn seed extract and fraction b (isolated from raw seeds) had a very slight genotoxic effect on PBMC cells. This effect was not significant in the roasted seed extract, which suggests that thermal processing could have decreased the genotoxicity of the seeds. Thermal processing can sometimes reduce the toxicity of plant preparations, as was demonstrated by Kowalczewski et al. (2019), where thermal processing decreased the cytotoxic activity of potato juice toward NCM460 (normal human colon mucosal epithelial) cells, at the same time increasing its potency against intestinal cancer cells (HT-29 and Caco-2) (Kowalczewski et al., 2019). However, the fact that the damage that the raw sea buckthorn seed extract and fraction b caused to PBMC cells’ DNA was minimal makes it hard to draw definite conclusions. Raw sea buckthorn seed extract had some genotoxic effect on HL-60 cells as well; the same could be observed with fractions a from raw seeds and e, f, and g (from roasted seeds). Both sea buckthorn seed extracts, fractions a and b, isorhamnetin 3-*O*-β-glucoside-7-*O*-α-rhamnoside, and serotonin also had a small genotoxic effect on A549 cells. Raw seed extract and fraction a (which contained mostly triterpenoid saponins) significantly increased the percent of tail DNA in all three cell types, indicating that it might possess genotoxic activity to a small degree. However, even though there were significant differences between groups, they were minimal and thus might not be biologically meaningful.

The present work also investigated the effects of two extracts (from raw and roasted seeds), seven fractions (three fractions (a, b, and c) from raw sea buckthorn seeds and four fractions (d, e, f, and g) from roasted sea buckthorn seeds), and two chemical compounds (isorhamnetin 3-*O*-β-glucoside-7-*O*-α-rhamnoside and serotonin) on the induction of apoptosis in human PBMC cells and selected cancer cell lines (HL-60 and A549). Apoptosis is a complex form of programmed cell death acting through multiple molecular pathways; caspase 3/7 activation is a well-recognized marker of this process (Riss et al., 2021). So far, only a few papers have described the effect of preparations from various parts of sea buckthorn on apoptosis (Farheen et al., 2024; Hibasami et al., 2005; Marciniak et al., 2021). Marciniak et al. (2021) reported that low-polarity fractions from sea buckthorn fruits induced apoptosis in HT29, PC3, and AGS cells after 24 and 48 h of incubation, at the concentrations equaling to IC_50_ and 2 × IC_50_. The occurrence of apoptosis was assessed by measuring caspase 3/7 activity and microscopic analysis of cell morphology coupled with dual staining with acridine orange and ethidium bromide (Marciniak et al., 2021). Sea buckthorn berry polyphenols (200 μg/mL) had a strong pro-apoptotic activity on MDA-MB-231 (human breast cancer) cells after 24 h incubation. After the treatment, approximately 69% of cells were apoptotic, which was measured by Annexin V and propidium iodine staining (Farheen et al., 2024). In another study, six polyphenols (syringetin, isorhamnetin, quercetin, pentamethylquercetin, kaempferol, and myricetin) isolated from sea buckthorn root were tested for their ability to induce apoptosis in leukemia cells. After 3 days of treatment, 40 μM of kaempferol, quercetin, and myricetin (but not isorhamnetin) induced apoptosis in HL-60 cells, which was assessed by the observation of morphological changes and DNA fragmentation (Hibasami et al., 2005). So far, no papers appear to have studied the effect of preparations from roasted sea buckthorn seeds on apoptosis. The used plant extracts, fractions, and pure chemical compounds exhibited different effects on this process. For the first time, we showed that fraction b (from raw sea buckthorn seeds) significantly increased apoptosis levels in HL-60 cells after 24 h of incubation. This is consistent with the results of the resazurin assay, where fraction b significantly reduced the viability of HL-60 cells, indicating that apoptosis played a part in the cytotoxic activity of fraction b. However, the raw and roasted extracts, isorhamnetin 3-*O-*β-glucoside-7-*O*-α-rhamnoside, and serotonin did not increase caspase 3/7 levels. The fact that isorhamnetin 3-*O-*β-glucoside-7-*O*-α-rhamnoside did not induce apoptosis in HL-60 cells is consistent with the results of Hibasami et al. (Hibasami et al., 2005). Apart from isorhamnetin glycosides, fraction b also contains quercetin, kaempferol, and their derivatives. Other authors have reported that quercetin can cause apoptosis in HL-60 cells by inhibiting the expression of phosphoinositide 3-kinase (PI3K), as well as Bax and caspase 2 and 3 activation, suggesting that quercetin and its derivatives might be partially responsible for the pro-apoptotic activity of fraction b. Quercetin also decreased HL-60 cells' proliferation through the blockade of the G_0_/G_1_ phase (Shen et al., 2003; Yuan et al., 2012). A slight increase in apoptosis levels could be observed in the extract from raw seeds, but the effect was not significant. This indicates that sea buckthorn seeds might contain compounds that can reduce viability by other cell death mechanisms or reduce proliferation without causing cell death. Interestingly, the caspase 3/7 activity in PBMC cells was reduced by both serotonin and fraction c (a serotonin-containing fraction from raw sea buckthorn seeds). Together with the fact that fraction c and serotonin decreased PBMC viability, decreased caspase 3/7 levels might suggest that the preparations from sea buckthorn seeds reduced PBMC cell proliferation without killing them, suppressed their metabolic activity, or caused cell death by other mechanisms. The fact that neither fraction c nor serotonin induced DNA damage in PBMC cells further supports the conclusion that reduced level of resazurin conversion might be due to arrested proliferation or slowed down metabolism.

ROS can play a dual role in cancer treatment. On one hand, cancer cells generate large amounts of ROS, which facilitate proliferation, invasion, migration, angiogenesis, and drug resistance. Due to this, various antioxidant compounds have been examined for their potential as anticancer agents. However, after crossing a certain threshold, ROS can cause significant oxidative damage to cancer cells, leading to cell death (Brandl et al., 2025). In a study by Farheen et al. (2024), 200 μg/mL sea buckthorn berry polyphenols decreased ROS generation in MDA-MB-231 cells by 88% (Farheen et al., 2024). Here, we observed that different preparations from sea buckthorn seeds had varying effects on ROS generation. On one hand, extracts from raw and roasted seeds, fraction b and fraction c, decreased ROS generation in HL-60 cells. On the other hand, fraction g, isorhamnetin 3-*O*-β-glucoside-7-*O*-α-rhamnoside, and serotonin significantly increased ROS generation in all cell types. Raw and roasted seeds, fraction c, and fraction f also enhanced the levels of ROS in PBMC cells. 50 μg/mL serotonin had the strongest effect on all cell types. These results are consistent with a study by Bianchi et al. (2005), where serotonin increased intracellular hydrogen peroxide production in rat myocytes (Bianchi et al., 2005). Fang et al. (2013) also reported that serotonin generated ROS in the murine hypothalamus through an NADPH oxidase-dependent pathway (Fang et al., 2013). In PBMC and HL-60 cells, isorhamnetin 3-*O*-β-glucoside-7-*O*-α-rhamnoside and serotonin increased ROS generation and reduced resazurin conversion, which might suggest that ROS caused damage that reduced the viability of these cells. At the same time, serotonin reduced apoptosis levels in PBMC, which means that the results of the resazurin assay were not due to apoptosis. Moreover, the increase in ROS generation by fraction c in PBMC cells is reflected in decreased conversion of resazurin, which might also suggest that ROS-damaged PBMC cells reduced their viability. Apoptosis levels were decreased by fraction c as well. However, we found that not all significant changes in ROS generation were clearly linked to the results of apoptosis or resazurin assays. For example, increased ROS generation caused by fraction g did not cause any visible changes in the resazurin assay’s results.

## 5 Conclusion

The present paper broadens the current state of knowledge on the biological activities of sea buckthorn seeds *in vitro,* providing novel insights into the toxicity of two extracts (from raw and roasted seeds), seven fractions (three fractions (a, b, and c) from raw sea buckthorn seeds and four fractions (d, e, f, and g) from roasted sea buckthorn seeds), and two chemical compounds (isorhamnetin 3-*O*-β-glucoside-7-*O*-α-rhamnoside and serotonin) in blood platelets, PBMC, HL-60, and A549 cells. Both extracts had similar effects – they slightly decreased the viability of blood platelets and HL-60 cells (but did not damage PBMC cells), caused a very small amount of DNA damage in all tested cells, and modified ROS levels in PBMC and HL-60. Fractions a and d (containing mostly triterpenoid saponins) had almost no effect on all the tested parameters. Fraction b was the most promising out of all the tested sea buckthorn seed preparations. It decreased the viability of HL-60 cells at 50 and 100 μg/mL, and increased their apoptosis levels. It was safe to blood platelets, but caused some damage to PBMC cells; however, the damage to PBMC was weaker than in HL-60 cells, as it was significant only at 100 μg/mL. Fraction b also decreased ROS levels in HL-60 cells. Fraction e, containing the same class of compounds as fraction b (mostly flavonol glycosides, including isorhamnetin 3-*O*-β-glucoside-7-*O*-α-rhamnoside), but isolated from roasted seeds, did not show such promising effects, indicating that thermal processing might have decreased its biological activity. Fractions c and f (containing serotonin, nucleosides, aromatic, amino acids, and B-type proanthocyanidins) had some unfavorable effects on PBMC cells – fraction c lowered the levels of conversion of resazurin to resorufin and decreased apoptosis, indicating either a toxic effect, reduced proliferation, or decreased metabolic activity. It also significantly increased ROS production in PBMCs, which might be the reason for the results of the resazurin assay. It also increased the viability or metabolic activity of A549 cancer cells. Its effects were similar to those of serotonin, indicating that this compound might be partially responsible for its activity. Fraction f (isolated from roasted seeds) had similar effects to fraction c. These findings indicate that fractions c and f shouldn’t be considered as potential anticancer agents. Fraction g increased the level of ROS and had small genotoxic activity in all cells, but this did not result in cytotoxic activity measured by the resazurin assay. Isorhamnetin 3-*O*-β-glucoside-7-*O*-α-rhamnoside decreased resazurin conversion in both PBMC and HL-60 cells to a similar degree. It did not induce apoptosis, but increased ROS generation in all cells. It did not damage blood platelets or increase the viability of cancer cells, but its effectiveness as an anti-cancer drug was not spectacular. Fraction b performed the best among all the tested extracts, fractions, and chemical compounds. In conclusion, fraction b from raw sea buckthorn seeds showed anticancer potential against HL-60 cells, but it should be studied further to determine the activity of individual compounds contained within, their mechanisms of action, and efficiency *in vivo*.

## Data Availability

The raw data supporting the conclusions of this article will be made available by the authors, without undue reservation.

## Glossary

5-HT: serotonin
A549: human lung adenocarcinoma
AGS: gastric adenocarcinoma cells
B16F10: mouse melanoma cells
Bax: Bcl-2-associated X protein
CaCo-2: colorectal adenocarcinoma cells
*CAT*: catalase gene
CPDA: citrate/phosphate/dextrose/adenine
DAPI: 4′,6-diamidino-2-phenylindole
DCF: 2′,7′-dichlorofluorescein
*GPX1*: glutathione peroxidase 1 gene
*GSR*: glutathione reductase gene
**H**_**2**_ **DCFDA**: 2′,7′-dichlorofluorescin diacetate
HBSS: Hanks' Balanced Salt Solution
HCT116: colorectal carcinoma cells
HFF-1: non-tumorous human fibroblasts
HL-60: human acute promyelocytic leukemia
HT29: colorectal adenocarcinoma cells
LDH: lactate dehydrogenase
LMP: low melting point
MCF-7: breast adenocarcinoma cells
MDA-MB-231: breast adenocarcinoma cells
NK: natural killer cells
NMP: normal melting point
*NQO1*: NAD(P)H dehydrogenase quinone 1 gene
Nrf2: nuclear factor erythroid 2-related factor 2
NSCLC: non-small cell lung cancer
PBMC: peripheral blood mononuclear cells
PC3: prostate adenocarcinoma cells
PI3K: phosphoinositide 3-kinase
**PLP**_**2**_: pig liver primary culture cells
ROS: reactive oxygen species
TRIB3: Tribbles pseudokinase 3
VERO: African green monkey kidney cells
